# Reproducibility of BiliCare™ Transcutaneus Bilirrubin Meter in Mexican Newborns

**DOI:** 10.1155/2019/3812152

**Published:** 2019-01-01

**Authors:** David Oldak, Gabriela García, Estela E. Gonzalez, Enver Aillon, Juan C. Falcon, Ernesto Ayala, Bernardo Oldak

**Affiliations:** ^1^Division of Neonatology, Department of Pediatrics, Angeles de las Lomas Hospital, State of México, Mexico; ^2^Health Science Department, School of Medicine. Anahuac University State of México, Mexico

## Abstract

**Background:**

Newborn hyperbilirubinemia is considered a worldwide health problem that demands medical evaluation. Noninvasive transcutaneous bilirubin (TcB) has been used as a screening method with different devices but there has not been any evaluation of reproducibility of the same brand devices. The BiliCare™ system is evaluated to demonstrate consistency between measurements with four different devices.

**Methods:**

336 TcB measurements were obtained with four BiliCare™ devices in 21 Mexican icteric newborns with a mean postnatal age of 44.1 hours of life and 38 weeks of gestation (33–41). Two measurements were taken in the same ear alternatively at the scaphoid fossa with each device. TcB values were compared between devices. Validity was compared with total serum bilirubin (TB).

**Results:**

intraclass correlation coefficient demonstrates a minimum limit in the study of 0.945 and maximum of 0.988 with the same device. Correlations with serum and between devices gave results above 0.932.

**Conclusions:**

BiliCare™ transcutaneous bilirubin measurement instrument has very good intra- and interdevice reproducibility; also correlation of TcS with serum bilirubin gave statistically the same results.

## 1. Introduction

Neonatal jaundice or yellow skin color in the newborn is a common physiological adaptation most often caused by the increased production of indirect bilirubin. Although it is generally harmless, some neonates may develop very high levels of unconjugated bilirubin [1, 2]. Jaundice requires medical evaluation since high levels (above 95th percentile for age in hours) of total serum bilirubin (TSB) that occurs in 5% to 6% of newborns is associated with encephalopathy or kernicterus, with concomitant irreversible central nervous system damage [3].

To decide if a child is in danger due to hyperbilirubinemia, the gold standard is the measurement of TSB. However, this procedure is invasive and often requires a blood sample of at least 0.5 ml, which for a newborn weighing 2.5 kg means 0.25% of his/her blood volume, and a laboratory capable of blood processing must be available. Although the risk associated with blood sampling is considered low, it does not exclude the possibility of complications, among which are the following: infection at the puncture site, bacteremia, osteomyelitis, and in repetitive sampling iatrogenic anemia [4]. Once results are obtained, bilirubin levels are stratified in specific nomograms according to the risk, gestational age, and hours of birth [5].

Since 1980, prototypes capable of estimating transcutaneous bilirubin (TcB) based on spectrophotometry have been developed; most of them use light absorbance by bilirubin, which is directly proportional to the concentration in subcutaneous cellular tissue [6, 7]. Furthermore, different types of devices called “second and third generation” have been created, which add various adjustments to eliminate cutaneous chromatophores such as melanin or hemoglobin.

Some of these devices have already been validated even in shielded skin and in children with phototherapy [8, 9].

They have been effective in detecting at-risk patients and helping on the decision to initiate treatment in order to decrease the risk of neurological damage or readmission for hyperbilirubinemia [10, 11].

The BiliCare™ a noninvasive bilirubin meter is a transcutaneous device that uses transmission technology instead of reflection. It adapts with an installed clip into the lobe of the neonate's ear as a location tissue for its measurement. It emits light at two wavelengths above the ear and measures the transmittance of light as it passes through the tissue, as the bilirubin molecules absorb light at a specific wavelength. The light source comes from two semiconductor emitting diodes (LED), one blue and one green. A high precision circuit controls the transmittance and calculates the bilirubin level in an efficient way without the need to correct the measurement with estimated formulas and without calibration prior to measurement as with other devices. BiliCare™ does not use consumables or spare parts.

To date, there is limited data to analyze both the accuracy and precision of the BiliCare™ device in clinical practice [12]. No study has been performed in order to verify differences among different devices of the same brand. The efficacy of this device was established in two unpublished studies sponsored by Natus, the manufacturer of BiliCare. The first one at the Soroka University in Israel where 75 patients with 36 weeks of gestation (WGA) or more were studied. The results showed that, using a cut-off point of <11mg/dL and >12mg/dL to start treatment, sensitivity was 94% and 80%, respectively, with 99% specificity and a negative predictive value of more than 98% in both cut-off points [13].

The second study conducted in Chita, Russia, included 144 patients of whom 107 were older than 35 WGA and 37 had between 25 and 35 WGA. They did not found alterations on BiliCare™ results in relation to gestational age, nor to skin color (p <0.0001), with a correlation coefficient of 0.93 [7, 14].

Grabenhenrich et al. compared serum and transcutaneous bilirubin measurements (using a bilirubin meter JM-103), before, during, and after phototherapy, in which 86 patients were included (47 term and 39 preterm). One hundred and eighty-nine measurements were made and it was found that the difference between serum and TcB samples before phototherapy was -0.6mg/dL (±1.9mg/dL) and during treatment the correlation increased as time elapsed in phototherapy. At 8, 16, and 24 hours of treatment, it was -1.8, -1.1, and -0.8mg/dL, respectively. The authors concluded that it is a very useful tool and explained that the variability occurs with the treatment since skin bilirubin lowers first with phototherapy but blood and skin concentrations pair up at 18-24 hours after the start of phototherapy [15]. It is not known if the weight at birth, hours of life, and gestational age at birth interfere with the validity of the measurement, and with the results obtained [16, 17].

BiliCare™ bilirubin meter has shown a high correlation coefficient above 0.90 with a statistical significance of p <0.001 in two previous studies. It offers advantages such as the absence of consumables and the LED technology that is currently the only one with these characteristics on the market [18, 19].

Many nurseries follow the recommendation to screen all infants with TcB especially before discharge. Guidelines and recommendations are constantly changed in many countries [20, 21]. The objective of the study is to address if there is any difference in measurement between BiliCare™ devices [22].

## 2. Material and Methods

An observational, single center, cross-sectional, analytical study was carried out at Hospital Angeles Lomas (State of México, México). The Investigation and ethics committee from the Anahuac University and Hospital Angeles previously approved the study. Informed consent was signed by all parents.

The sample size was calculated using the Zou formula [23] with correlation coefficient and a power of 80% and 92.72 empirical assurance. All results were obtained using Pearson correlation and the intraclass correlation coefficient (ICCA2) according to the nature of the variables; all data was analyzed using SPSS statistics (IBM, Chicago).

Icteric newborn patients admitted to the nursery, intermediate neonatal care, and intensive neonatal care with no other disease and which by their condition and according to national and international guidelines TSB was indicated were included [4]. Patients receiving phototherapy before sampling were excluded.

Blood samples were taken by venous puncture; all were analyzed and processed in Architect c16000 Systems (Abbott Lab) (ARCHITECT TOTAL BILIRRUBIN B6L4U3, DIRECT BILIRRUBIN B8G6W3). All samples were processed in duplicate in order to discard laboratory discrepancy. TcB readings were performed randomly by two skilled operators involved with jaundice assessment; instruments were calibrated according to the manufacturer's instructions.

While the sample was processed in the laboratory (±30 minutes of blood withdrawal) and without knowing the results, the child was placed with the head facing up for at least 5 minutes in order to avoid different blood flow at the earlobes by pressure with the mattress. The BiliCare clip was located on baby's Scaphoid Fossa and 4 TcB measurements were taken alternating ears of each patient with four different BiliCare devices (total 16 determinations per patient).

## 3. Results

21 patients with clinical jaundice were studied, all of them without past phototherapy with a mean age of 44.1 hours of extrauterine life. 13 from the 21 patients were males (61.9%). The average WGA was 38 (±2.22) most of them being full term (90.48%) and with normal weight for the gestational age (85.71%) (Table 1). All 21 patients were assessed.

First, duplicate values of serum bilirubin were correlated to obtain the reproducibility of the standard technique obtaining an ICCA2 of 0.976 (95% CI = 0.940-0.990) with a variation of the mean of 0.327 mg/dl between groups.

TcB measurements for each patient were compared with values of paired serum samples obtaining an ICCA2 of 0.98 (95% CI = 0.969-0.995). Furthermore, each device average was compared with the mean of paired serum samples, obtaining ICC2A1 values from 0.943 for device 1 to 0.964 for device 3 (Table 2).

In Table 3 two measurements were made on each ear for each patient obtaining values above 0.9 meaning that the device is consistent between measurements. As well correlations were made to assure that all devices were giving the same values all of them being above 0.9. The ICCA2 demonstrates a minimum limit of 0.945 comparing device 3 in the left ear and a maximum of 0.988 in 3 different devices (Table 3).

To compare reproducibility between devices, the mean of the 4 samples taken by each device (two on the right ear and two on the left) were compared with the mean; the other devices ICC2A1 of 0.932 for device 1 versus device 3 were the smallest intraclass correlation (Table 4).

## 4. Discussion

Unreliable measurements can have serious consequences in medical practice. TcB measurement is commonly used to assess bilirubin levels in neonates, showing their effectiveness in detecting at-risk patients and the need to initiate treatment. TcB is used as a screening method for hyperbilirubinemia. Systematic reviews [6, 24] concluded that TcB devices reliably estimated bilirubin levels in term and preterm infants but none of the actual guidelines suggests the use of TcB measurements as an indication for starting phototherapy.

We used the intraclass correlation coefficient (ICCA2) because it is defined as the proportion of all variation that is not due to measurement error [23]. The interpretation of ICCA2 (p) is facilitated using the benchmark that p<0 reflects “poor” reliability and above 0.81 “almost perfect” [25].

As correlation between the four devices and serum samples obtained values higher than 0.9 showing that in these patients having no phototherapy TSB values are as reliable as serum measurements for therapeutic decisions.

Another essential factor to use these techniques for therapeutic decisions is knowing that the device is measuring the same all the time.

Since all correlations were in the “almost perfect” area of correlation, the BiliCare™ is a reliable device not only for bilirubin screening but also as a fast and noninvasive tool for therapeutic decisions in this type of newborns who have not been exposed to sunlight or phototherapy.

As clinicians, we have the need to use new technologies since in daily practice we face discrepancies between tests. Most investigators have compared different bands of transcutaneous devices but only a few studies have been performed in order to evaluate the accuracy of the same brand and model of instruments in the same patient. Regarding Bilicare™, a previous study revealed no difference in between examiners [19]. This article demonstrates that serum bilirubin determination in our media (tertiary hospital in Mexico) and with newborns not exposed to light of phototherapy has a correlation coefficient of 0.98 (only 2% error) in comparison with Bilicare™ transcutaneous bilirubin measurement instrument and it has a very good reproducibility, greater than 0.93 in all the statistical tests to which it was subjected.

## 5. Conclusions

Results show that all BiliCare™ instruments measure statistically the same in comparison with serum, between repeated measurements and different devices.

This device could be considered a very useful instrument, noninvasive, without risks for the patient, and without the need for training for its use. Results can be compared between hospitals or even countries because of its accuracy. We do not know how the device behaves in patients undergoing phototherapy and further studies must be done to demonstrate its utility in different newborn races and gestational age.

## Figures and Tables

**Table 1:** Demographic Characteristics n=21.

Gender		n (%)
	Male	13 (61.90)
	Female	8 (38.10)

Department		
	Nursery	13 (61.90)
	Intermediate Care	6 (28.57)
	Intensive Care	2 (9.52)

Newborn Classification		
	Full term	
	Normal weight	16 (76.19)
	Low weight	3 (14.29)
	Preterm	
	Normal weight	1 (4.76)
	Postterm	
	Normal weight	1 (4.76)

**Table 2:** Correlation between BiliCare™ and serum bilirubin.

		**Serum**
**Device 1**	ICC2A1	0.943
	Pearson	0.954

**Device 2**	ICC2A1	0.966
	Pearson	0.959

**Device 3**	ICC2A1	0.964
	Pearson	0.961

**Device 4**	ICC2A1	0.954
	Pearson	0.964

**Table 3:** BiliCare™ intra-apparatus assay.

		**Right ear 1 VS 2**	**Left ear 1 VS 2**
**Device 1**	ICC2A1	0.981	0.988
****	Pearson	0.963	0.975

**Device 2**	ICC2A1	0.988	0.98
****	Pearson	0.977	0.965

**Device 3**	ICC2A1	0.979	0.945
****	Pearson	0.958	0.896

**Device 4**	ICC2A1	0.987	0.988
****	Pearson	0.978	0.977

**Table 4:** BiliCare™ interapparatus assay.

		**Device 1**	**Device 2**	**Device 3**	**Device 4**
**Device 1**	ICC2A1	1	0.980	0.932	0.982
	Pearson	1	0.973	0.945	0.964

**Device 2**	ICC2A1	0.980	1	0.957	0.983
	Pearson	0.973	1	0.943	0.973

**Device 3**	ICC2A1	0.932	0.957	1	0.955
	Pearson	0.945	0.943	1	0.973

**Device 4**	ICC2A1	0.982	0.983	0.955	1
	Pearson	0.964	0.973	0.973	1

## Data Availability

The data used to support the findings of this study are available from the corresponding author upon reasonable request.
